# Genetic Determinants of Chronic Obstructive Pulmonary Disease in South Indian Male Smokers

**DOI:** 10.1371/journal.pone.0089957

**Published:** 2014-02-24

**Authors:** Cholendra Arja, Rajasekhara Reddy Ravuri, Venugopal N. Pulamaghatta, Krishna Mohan Surapaneni, Premanand Raya, Chandrasekhar Adimoolam, Kodanda Reddy Kanala

**Affiliations:** 1 Department Of Anthropology, Division Of Human Genetics, Sri Venkateswara University, Tirupati, Andhra Pradesh, India; 2 Department Of Human Genetics, Punjabi University, Patiala, Punjab, India; 3 Anthropological Survey Of India, Southern Regional Centre, Mysore, Karnataka, India; 4 Department Of Biochemistry, Saveetha Medical College & Hospital, Faculty Of Medicine, Saveetha University, Chennai, Tamil Nadu, India; 5 Premananda Allergy And Chest Hospital, Tirupati, Andhra Pradesh, India; Sanjay Gandhi Medical Institute, India

## Abstract

The development of chronic obstructive pulmonary disease, upon exposure to tobacco smoke, is the cumulative effect of defects in several genes. With the aim of understanding the genetic structure that is characteristic of our patient population, we selected forty two single nucleotide polymorphisms of twenty genes based on previous studies and genotyped a total of 382 samples, which included 236 patients and 146 controls using Sequenom MassARRAY system. Allele frequencies of rs2276109 (MMP12) and rs1800925 (IL13) differed significantly between patients and controls (p = 0.013 and 0.044 respectively). Genotype analysis showed association of rs2276109 (MMP12) under additive and dominant models (p = 0.017, p = 0.012 respectively), rs1800925 (IL13) under additive model (p = 0.047) and under recessive model, rs1695 (GSTP1; p = 0.034), rs729631, rs975278, rs7583463 (SERPINE2; p = 0.024, 0.024 and 0.012 respectively), rs2568494, rs10851906 (IREB2; p = 0.026 and 0.041 respectively) and rs7671167 (FAM13A; p = 0.029). The minor alleles of rs1695 (G), rs7671167 (T), rs729631 (G), rs975278 (A) and rs7583463 (A) showed significant negative association whereas those of rs2276109 (G), rs2568494 (A), rs10851906 (G) and rs1800469 (T; TGF-β) showed significant positive association with lung function under different genetic models. Haplotypes carrying A allele of rs2276109, G allele of rs1695 showed negative correlation with lung function. Haplotypes carrying major alleles of rs7671167 (C) of FAM13A and rs729631 (C), rs975278 (G), rs7583463 (C) of SERPINE2 had protective effect on lung function. Haplotypes of IREB2 carrying major alleles of rs2568494 (G), rs2656069 (A), rs10851906 (A), rs965604 (C) and minor alleles of rs1964678 (T), rs12593229 (T) showed negative correlation with lung function. In conclusion, our study replicated the results of most of the previous studies. However, the positive correlation between the minor alleles of rs2568494 (A) and rs10851906 (G) of IREB2 and lung function needs further investigation.

## Introduction

The spectrum of Chronic Obstructive Pulmonary Disease (COPD) involves two pathological conditions, chronic bronchitis and emphysema. In India, tobacco smoke and exposure to biomass fuel exhaust (animal dung cakes, wood and crop residues) are the risk factors in males and females respectively [1]. In India an estimated 82.3% of males with COPD are smokers [2]. According to the estimation of World Health Organization (WHO) in 2002, COPD was responsible for over half a million deaths in India, placing the country second in the world in COPD mortality [3].

Alpha 1-antitrypsin deficiency is the only established genetic factor that invariably results in COPD in smokers. However, this is found only in about one percent of cases [4]. Linkage studies and subsequent association studies carried out with positional candidate genes or genes selected based on their presumptive role in pathophysiology have identified numerous genetic markers falling into the classes of antioxidants, proteases, antiproteases and inflammatory mediators [5]. However, the replication of genetic associations across different populations has not always been consistent [6]. While sample size, subject selection criteria and study designs contribute their share, the complex interplay between various molecules that are involved in the maintenance of lung integrity, which makes COPD polygenic, renders consistency across different population settings rather unlikely. Nevertheless, such studies helped us understand the pathogenesis of COPD under the headings of oxidant-antioxidant imbalance theory, the protease-antiprotease imbalance theory and inflammation. This genetic complexity and hence the pathophysiological heterogeneity together with the variability attributed to the disease by the environment, rendered COPD an incurable disease so far.

Identifying a common pathway(s) that links exposure to emphysema is possible only when genes implicated in the pathogenesis of COPD in one population are validated in other populations. To this end we selected forty two SNPs across twenty genes by referring to previous studies on COPD to identify the genetic makeup that is signature of our patient population.

## Materials and Methods

### Subjects

A total of 386 males (236 patients and 150 controls) were included in the study. All subjects were *bidi* (tobacco wrapped in tendu leaf (*Diospyros melanoxylon*)) smokers and were over 40 years of age with a smoking history >10 pack years. COPD diagnosis and staging was done using GOLD criteria [7]. Spirometry was performed while the patients were in stable condition using SpiroWin Model No. 99 spirometer (Genesis Medical Systems, Hyderabad, Andhra Pradesh, India.). All patients were requested to withhold their COPD medications for six hours (for short acting bronchodilators) or twelve hours (for long acting bronchodilators). Patients were required to have a post FEV_1_/FVC ratio <70%. Subjects with a history of lung cancer, bronchial asthma, bronchiectasis, cystic fibrosis and fibrosis of pulmonary tuberculosis were excluded from the study. Patients were requested to stop all the medications they were using for a period of 24 hours prior to the day of testing. Reversibility of air flow obstruction was tested within 10–15 min after administering 0.5% salbutamol nebulizer solution at a dosage of 0.02 ml/kg body-weight diluted to 2 ml with isotonic saline with a compressed air driven nebulizer (Pulmo-Aide 5610, Devilbiss; DeVilbiss Healthcare, Somerset, PA, USA). Patients who showed reversibility ≥12% predicted and ≥200 ml of the absolute value of FEV_1_ were excluded from the study.

While patients were available at the clinic, controls matching patients for age, smoking medium and pack years had to be searched for and could be reached only at their work places. Hence a portable spirometer (Vitalograph copd-6; Vitalograph Inc., Lenexa, KS, USA) which gave FEV_6_ was used to diagnose controls. Prior to use with controls, the portable spirometer was tested against the standard spirometer at the clinic to assess the validity of the former’s readings. Apparently normal individuals, strictly with an FEV_1_/FEV_6_ ratio >70% were selected as controls. Irrespective of the spirometry values, subjects were excluded from the control group if they reported difficulty in breathing while walking or working at any point of time in their life, have/had exposure to risk factors other than smoking (biomass fuel exhaust, diesel exhaust, grain dust, cotton dust), ceased to smoke at any point of time in their life due to breathing problems or visited any physician due to respiratory problems. A written informed consent was obtained from all the subjects prior to their participation in the study. The study protocol was approved by the Human Ethics Committee of Sri Venkateswara University (No:SVUHEC/ANTH/2010-09).

### SNP Selection and Genotyping

Forty two SNPs from twenty genes were selected based on previous studies on COPD (Table S1). Genomic DNA was extracted from about 10 ml of whole peripheral blood using standard phenol-chloroform method. All subjects were genotyped using Sequenom’s MassARRAY system (Sequenom Inc., San Diego, California, USA) according to manufacturer’s specifications for the iPlex chemistry using 10 ng genomic DNA. Prior to further analysis, the assay performance and genotype calls were qualified by evaluating genotype cluster plots.

### Statistical Analyses

Descriptive statistics were calculated using SPSS v16.0 (SPSS Inc., Chicago, IL, USA). Discontinuous variables are presented with percentages. Mean and standard deviation were calculated for clinical characteristics and compared between patients and controls using unpaired Student’s *t-*test after adjusting for age, pack years and age - pack years interaction. Genetic analyses were carried out using PLINK software [8]. All the SNPs were checked for deviation from Hardy-Weinberg equilibrium in controls. Allele frequency variations were compared between patients and controls using Pearson’s Chi-square test to generate odds ratio with 95% confidence limits. The contribution of each genotype to COPD susceptibility was evaluated using logistic regression under additive, dominant and recessive genetic models after adjusting for age and pack years. A linear regression model was used to study the association of SNPs with two COPD phenotypes (FEV_1_ and FEV_1_/FVC) under three genetic models with age and pack years as covariates. Haplotypes were generated using a sliding window method and their association was tested against COPD and its phenotypes using regression model after adjusting for age and pack years. The sliding window approach implemented in PLINK sequentially examines smaller sets of SNPs within the region. For example, using a 4-SNP overlapping sliding window, one would first conduct a haplotype analysis of SNPs 1–4, followed by SNPs 2–5, followed by SNPs 3–6, and so on until the last SNP in the region is reached [9], [10]. A p value less than 0.05 was considered as significant throughout the analyses. The Benjamini–Hochberg False Discovery Rate method was used to correct for multiple hypothesis testing for allele and genotype association [11], whereas maxT permutation of 10000 steps was used to generate adjusted empirical p value for haplotype association tests [8].

## Results

Demographics and clinical characteristics of the study population are presented in table 1. The age of the study population ranged from 40–80 years. Most of the subjects were older than 60 years (69.5% in patients, 55.5% in controls). There were more patients (42.4%) with BMI <18.5 kg/m2 compared to controls (17.9%). The majority of patients and controls were heavy smokers (69.06% and 74.7% respectively). The smoking intensity (calculated as pack years) was greater in control group than in patients (48.24±25.76 vs. 42.96±23.53; p = 0.03). GOLD COPD staging identified most of the patients in stages III and IV (44.1% and 39.4% respectively).

**Table 1:** Demographics and clinical characteristics of the patients and controls.

		COPD (n = 236)	Controls (n = 150)	*t*-value	*p*-value
		Mean (SD)	Mean (SD)		
**Age**		63.19 (8.79)	61.07 (9.05)	2.272	0.02
**Pack years®** ¥		42.96 (23.53)	48.24 (25.76)	2.01	0.03
**BMI** ‡		19.82 (3.84)	22.01 (3.17)	6.04	0.07
**FEV1%** ‡		36.82 (13.67)	71.68 (11.77)	26.42	0
**FEV_1_/FVC** ‡		55.42 (9)	85.98* (7.67)	35.37	0
**Occupation (%)**	Agriculture	56.8	52.1		
	Labor	13.6	17.8		
	Business	14.8	8.2		
	Employees	14.8	21.9		
**GOLD COPD staging (%)**	I	0.8			
	II	15.7			
	III	44.1			
	IV	39.4			

® p - value adjusted for age.

‡ p-value adjusted for age and pack years.

*FEV_1_/FEV_6_.

¥ Pack years were calculated by multiplying the number of bidis smoked per day with number of years of smoking, and then dividing the product by 24 (number of bidis in a packet) [42].

The SNPs genotyped and genes studied along with results of allelic association are presented in table S1. Four control subjects had insufficient DNA quality and had to be excluded. Hence 146 control samples were genotyped. None of the SNPs deviated significantly from Hardy-Weinberg equilibrium in controls (p>0.05). Two SNPs in SERPINA3 (rs1800463 and rs17473), which had minor allele frequency <0.01 were excluded from further analysis. The minor allele frequencies of two SNPs, one in MMP12 (rs2276109) and another in IL13 (rs1800925) differed significantly between patients and controls (OR = 0.476, 95% CI: 0.263–0.863, p = 0.013 and OR = 1.453, 95% CI: 1.009–2.091, p = 0.044 respectively). The significance was lost after correcting for multiple testing. Logistic regression analysis after adjustment for age and smoking history under different genetic models revealed association of MMP12 (rs2276109) under additive and dominant models (p = 0.017, p = 0.012 respectively), IL13 (rs1800925) under additive model (p = 0.047) and GSTP1 (rs1695, p = 0.034), SERPINE2 (rs729631, p = 0.024; rs975278, p = 0.024; rs7583463, p = 0.012), IREB2 (rs2568494, p = 0.026; rs10851906, p = 0.041) and FAM13A (rs7671167, p = 0.029) under recessive model (table S2). None of the SNPs retained significance after correction for multiple testing.

Among the SNPs genotyped, nine SNPs showed significant association with FEV_1_, and/or FEV_1_/FVC (Table 2). The T allele of FAM13A (rs7671167) showed significant negative association with FEV_1_ under additive and recessive models (p = 0.013 and 0.011 respectively) and with FEV_1_/FVC under recessive model (p = 0.036). The G allele of GSTP1 (rs1695) showed significant negative association with FEV_1_ under additive (p = 0.043) and recessive models (p = 0.001) and with FEV_1_/FVC under recessive model (p = 0.023). The association of rs1695 under recessive model with FEV_1_ remained significant even after correction for multiple testing (p-FDR = 0.026). The G allele of MMP12 (rs2276109) showed significant positive association with FEV_1_ under dominant model (p = 0.050) and with FEV_1_/FVC under additive (p = 0.015) and dominant models (p = 0.007). Two SNPs in IREB2 (rs2568494-A and rs10851906-G) showed marginal positive association with only FEV_1_ under recessive model (p = 0.052). The T allele of TGF-β showed association with FEV_1_/FVC under dominant model (p = 0.028). Three SNPs in SERPINE2 (rs729631-G, rs975278-A, rs7583463-A), showed significant positive association with both the phenotypes under recessive model (FEV_1_, p = 0.002; FEV_1_/FVC, p = 0.011, 0.011, 0.007 respectively). The p values remained significant after correction for multiple testing only for FEV_1_ (p-FDR = 0.026).

**Table 2:** Single nucleotide polymorphisms showing association with COPD phenotypes.

GENE	SNP ID	Allele	β- FEV_1_	P1*	Model#	P-FDR	β-FEV_1_/FVC	P2*	Model#	P-FDR
FAM13A	rs7671167	T	−3.913, −6.773	0.013, 0.011	A, R	0.302, 0.087	−4.436	0.036	R	0.290
GSTP1	rs1695	G	−3.425, −13.25	0.043, 0.001	A, R	0.694, 0.026	−7.184	0.023	R	0.220
MMP12	rs2276109	G	6.557	0.050	D	0.943	6.024, 7.095	0.015, 0.007	A, D	0.371, 0.359
SERPINE2	rs729631	G	−10.89	0.002	R	0.026	−7.195	0.011	R	0.133
SERPINE2	rs975278	A	−10.89	0.002	R	0.026	−7.195	0.011	R	0.133
SERPINE2	rs7583463	A	−9.523	0.002	R	0.026	−6.655	0.007	R	0.133
IREB2	rs2568494	A	9.445	0.052	R	0.275				
IREB2	rs10851906	G	6.524	0.052	R	0.275				
TGF –β	rs1800469	T					3.857	0.028	D	0.4467

P1*: p-value for FEV_1_ adjusted for age and pack years. P2*: p-value for FEV_1_/FVC adjusted for age and pack years.

P-FDR: p-value corrected for multiple hypothesis testing by Benjamini–Hochberg False Discovery Rate method.

# A: Additive, R: Recessive, D: Dominant.

Using a sliding window approach we generated haplotypes of 2, 3 and 4 SNPs and analyzed their association with COPD, FEV_1_ and FEV_1_/FVC. The haplotypes showing nominal significant association are presented in table S3. Haplotypes carrying the G allele of rs2276109 (MMP12) had a significant protective effect against developing COPD. Haplotypes of MMP12 carrying the A allele of both rs652438 and rs2276109 conferred significant risk of developing the disease. The IREB2 haplotypes containing the major alleles of rs2568494, rs2656069, rs10851906, rs965604 and minor alleles of rs1964678 and rs12593229 showed significant negative association with both COPD and lung function parameters. The SERPINE2 haplotypes containing major alleles of rs729631, rs975278, rs7583463 and minor allele of rs16865421 had a significantly greater frequency in controls and were positively associated with lung function. The two SNP haplotype of GSTP1 containing the minor allele G of rs1695 was negatively associated with FEV_1_. This effect appeared to be profound when in combination with the risk haplotype AA of MMP12. However, G allele of rs1695 did not seem to be sufficient enough to produce the detrimental effect when in combination with the protective haplotype AG of MMP12. The 2, 3 and 4 SNP haplotypes constructed out of SNPs of the genes studied on chromosome 4 (GC, FAM13A, HHIP), showed that the haplotypes carrying the C allele of FAM13A had a protective effect on lung function. There were more controls carrying the haplotype CTCA (rs4588-rs7041-rs7671167-rs1828591) than patients. The frequencies of the two SNP haplotypes of EPHX1 did not differ significantly between patients and controls (data not shown). However, the presence of EPHX1 haplotype carrying minor allele C of rs1051740 and G of rs2234922 was found to have a protective effect (β = 6.44, p = 0.037). None of the haplotypes retained their significance after adjusting for multiple testing.

## Discussion

In this study, we aimed at understanding the genetic structure that underlies the risk of developing COPD in our study population. To accomplish this, subjects were screened for single nucleotide polymorphisms of the genes falling into the classes of antioxidants, detoxification, proteases, antiproteases, inflammatory mediators and also those identified recently through GWAS. In agreement with the pathophysiological heterogeneity of the disease associations were found with the genes belonging to different classes.

MMP12 is an elastase which is predominantly produced by the alveolar macrophages. The lung tissues of the patients with advanced emphysema abound in MMP12 protein [12] and mice lacking MMP12 activity are protected against cigarette smoke induced emphysema [13]. The *A* allele of MMP12 SNP rs2276109 (−82 A→G) is associated with higher gene expression [14]. The functional impact of SNP rs652438 (1082A→G) on MMP12 activity is not known. In the present study, the frequency of rs2276109 *G* allele is significantly higher in controls. A significant positive correlation was also found between the rs2276109 G allele and FEV_1_ under dominant model (β = 6.557, p = 0.050) and FEV_1_/FVC under dominant (β = 7.095, p = 0.007) and additive models (β = 6.024, p = 0.015). Though the frequency of G allele of rs652438 was higher in controls, it did not reach significance level (0.061 in cases vs. 0.075 in controls, OR = 0.803, 95% CI: 0.452–1.427, p = 0.454). The deleterious effect of the A alleles of both rs2276109 and rs652438 is evident throughout the haplotype analysis. The frequency of AA haplotype was significantly higher in cases than in controls (0.894 and 0.836 respectively, p = 0.031). But the AA haplotype alone was not able to significantly decrease lung function (FEV_1_: β = −2.53, p = 0.26; FEV_1_/FVC: β = −3.27, p = 0.066 respectively). However 3 and 4 SNP haplotypes in which A allele of either SNP was present showed significant negative association with the lung function. Our result with respect to MMP12 is in agreement with previous studies [15], [16].

Studies in murine models showed that over expression of IL13 produces cathepsin and matrix metalloproteinase dependent emphysema, mucus metaplasia and inflammation [17]. The SNP rs1800925 (−1111 C→T) which results in increased production of IL13 [18] showed association with COPD in earlier studies [19], [20]. In our study too, the *T* allele of IL-13 showed significant association with the risk of developing COPD (OR = 1.453, 95% CI: 1.009–2.091, p = 0.044). In addition to this our genotype tests showed significant association of rs1800925 with COPD under additive genetic model (OR = 1.443, 95% CI: 1.005–2.071, p = 0.047).

Studies on animal models showed that decreased TGF- β signaling results in emphysema through alterations in macrophage MMP12 expression [21], [22]. The SNP rs1800469 of TGF- β (-509 C→T) is associated with increased expression [23]. Consistent with the physiological role of TGF- β in emphysema, earlier study [24] found association of *C* allele with COPD. In our study the *T* allele frequency was higher in controls, but the difference between patients and controls was not statistically significant (0.350 in cases vs. 0.404 in controls, OR = 0.793, 95% CI: 0.587–1.071, p = 0.129). However, in the regression analysis, the *T* allele showed a significant positive correlation with FEV_1_/FVC (β = 3.857, p = 0.028) under dominant model.

GSTs are a family of enzymes that catalyze the conjugation of reduced glutathione and subsequent detoxification of wide range of electrophilic substrates. Among the isoenzymes of GST, GSTP1 is the predominant form in human lung [25]. Two polymorphisms in GSTP1 have been investigated in COPD till date; rs1695 (A→G, Ile105Val,) and rs1138272 (C→T, Ala114Val). The replacement of Ile with less bulkier Val increases the catalytic activity of the enzyme towards polycyclic aromatic hydrocarbon diol epoxides [26], [27]. But contrary to what could be expected, this increased catalytic activity was found to be associated with several forms of cancer including that of lung [28], [29]. Studies in COPD with GSTP1 polymorphisms have shown mixed results. Some studies showed association of 105Ile variant with the disease [30], [31] while some showed association with 105Val variant [32], [33]. In our study, the SNP rs1695 showed association with COPD under recessive model (OR = 2.756, 95% CI: 1.081–7.026, p = 0.034). The rs1695 G allele showed significant negative correlation with FEV_1_ under recessive (β = −13.25, p = 0.001) and additive model (β = −3.425, p = 0.043) and with FEV_1_/FVC under recessive model (β = −7.184, p = 0.023). Significant negative correlations were also found between rs1695 G- rs1138272 C haplotype and FEV_1_ (β = −4.59, p = 0.010). The haplotypes carrying the A allele of rs1695 had significant positive effect on FEV_1_ (β = 3.43, p = 0.043).

FAM13A is a tumor suppressor gene. Earlier studies [34], [35] showed that the C allele of FAM13A rs7671167 (T→C) has a protective effect on COPD and our study supports the same. The frequency of T allele was greater in patients than in controls, but the difference was not significant (0.492 in cases vs. 0.432 in controls, p = 0.094). The SNP rs7671167 showed association with COPD under recessive model (OR = 1.853, 95% CI: 1.066–3.223, p = 0.029). The T allele also showed significant negative correlation with lung function (FEV_1_: β = −6.773, p = 0.011, recessive model; β = −3.913, p = 0.013, additive model) (FEV_1_/FVC: β = −4.436, p = 0.036, recessive model).

SERPINE2 is a member of serine protease inhibitor family and is capable of inhibiting thrombin, urokinase, plasmin and trypsin. Two major studies [36], [37], showed association of SERPINE2 polymorphisms with COPD. Another study [38] conducted in Japanese population showed association of rs975278 of SERPINE2 with emphysema under recessive model. In our study SNPs rs729631 (OR = 2.558, 95% CI: 1.131–5.788, p = 0.024), rs975278 (OR = 2.558, 95% CI: 1.131–5.788, p = 0.024), rs7583463 (OR = 2.407, 95% CI: 1.212–4.781, p = 0.012) of SERPINE2 showed significant association with COPD under recessive model. The same SNPs also showed significant negative correlation with FEV_1_ and FEV_1_/FVC under recessive model.

IREB2 together with IREB1 is involved in the regulation of cellular iron metabolism. Increased levels of IREB2 m-RNA have been reported in the lungs of smokers and COPD patients [39]. The polymorphisms of IREB2 have no known functional impact. Since the presence of excess iron in lung tissues can contribute to oxidative stress, abnormalities in IREB2 functioning or expression are likely to influence the pathology of COPD by augmenting oxidative stress. The minor allele frequency of all the IREB2 SNPs studied, with the exception of rs965604 was higher in controls than in cases. However, the difference was not significant. The SNPs rs2568494 and rs10851906 showed association with COPD under recessive model (OR = 0.336, 95% CI: 0.129–0.876, p = 0.026 and OR = 0.512, 95% CI: 0.270–0.972, p = 0.041 respectively). Further, rs2568494-A and rs10851906-G alleles showed marginal positive correlation with FEV_1_ (β = 9.445, p = 0.052 and β = 6.524, p = 0.052 respectively). The protective effect of rs2568494-A allele is contrary to earlier findings [39], [40], [41]. Further the major alleles rs1964678-C and rs12593229-G were reported to confer risk in a previous study [39] Contrary to this, the haplotypes carrying the minor alleles rs1964678-T and rs12593229-T were associated with the significant risk of developing the disease and showed significant negative correlation with lung function. The associations found with respect to IREB2 in our study cannot be considered conclusive and generalized to the population from which the sample was drawn because of our small sample size. Further, there is also a chance that these associations could be affected by the SNPs of nearby genes with which the IREB2 SNPs are in LD.

Our study has few limitations. Firstly, only male subjects were included in the study. This was due to lack of affected female subjects available under smoking category. Exposure to biomass fuel smoke (wood, cow dung cakes etc) is the predominant risk factor for COPD in females in India. Therefore only smokers were chosen with the assumption that the mechanism by which tobacco smoke, which is a carrier of several Group I and Group II carcinogens, initiates COPD could be different from that of biomass fuel smoke. Second limitation of our study is the sample size. One factor that greatly contributed to this was the strict adherence to *bidi* smokers. Cigarette is expensive than bidi. As most of the interviewed subjects were daily wage labors, the choice of smoking medium depended highly on the person’s day to day variable financial status. There were subjects who smoked both bidi and cigarette. Such subjects were excluded to avoid misinterpretation of pack years. Lastly, our patient population is not uniformly distributed across different GOLD stages of COPD. COPD was unknown to all our subjects until diagnosis (in case of patients) or our visit (in case of controls). Patients consulted physician only when they had severe respiratory problems due to disease progression. Therefore, at the time of initial diagnosis, most of the patients were either in GOLD stage III or GOLD stage IV.

## Conclusion

Our study managed to reinforce the theories of oxidant-antioxidant imbalance, protease-antiprotease imbalance and inflammation upon which the etiology of COPD has been built. While most of the associations found in this study have been reported elsewhere, the associations found with IREB2 need to be investigated with larger sample sizes.

## Supporting Information

Table S1 (DOCX)

Table S2 (DOCX)

Table S3 (DOCX)
