# Supplementary material for: Genetic Determinants of Chronic Obstructive Pulmonary Disease in South Indian Male Smokers
Source: PLoS One. 2014 Feb 24;9(2):e89957. doi: 10.1371/journal.pone.0089957 (PMC3933698; doi:10.1371/journal.pone.0089957)
Supplement: Table S1 — (DOCX) [file pone.0089957.s001.docx]

**Supplementary table S1**. Single nucleotide polymorphisms genotyped, Hardy-Weinberg equilibria and the results of allelic association.

| **GENE** | **SNP ID** | **ALLELES** | **MAF** | | **HWE**  **p-Value** | **OR (95%CI)** | **P** | **Reference** |
| --- | --- | --- | --- | --- | --- | --- | --- | --- |
|  |  | **minor/major** | **cases** | **controls** |  |  |  |  |
| EPHX1 | rs1051740 | C/T | 0.386 | 0.404 | 0.230 | 0.925 (0.687- 1.247) | 0.611 | 1 |
| EPHX1 | rs2234922 | G/A | 0.203 | 0.226 | 0.099 | 0.874 (0.614- 1.246) | 0.457 |  |
| SOD3 | rs8192287 | T/G | 0.136 | 0.127 | 0.470 | 1.081 (0.701- 1.669) | 0.725 | 2 |
| SOD3 | rs8192288 | T/G | 0.134 | 0.127 | 0.470 | 1.062 (0.687- 1.640) | 0.788 |  |
| SOD3 | rs1799895 | G/C | 0.066 | 0.051 | 1.000 | 1.298 (0.688- 2.448) | 0.419 |  |
| CAT | rs1001179 | A/G | 0.261 | 0.257 | 0.135 | 1.020 (0.731- 1.423) | 0.909 | 3 |
| GSTP1 | rs1695 | G/A | 0.303 | 0.274 | 0.059 | 1.152 (0.833- 1.592) | 0.392 | 4 |
| GSTP1 | rs1138272 | T/C | 0.044 | 0.062 | 0.428 | 0.709 (0.371- 1.354) | 0.295 |  |
| MMP12 | rs652438 | G/A | 0.061 | 0.075 | 0.182 | 0.803 (0.452- 1.427) | 0.454 | 5 |
| ***MMP12*** | ***rs2276109*** | ***G/A*** | ***0.044*** | ***0.089*** | ***1.000*** | ***0.476 (0.263- 0.863)*** | ***0.013**** |  |
| TIMP2 | rs2277698 | A/G | 0.275 | 0.274 | 0.407 | 1.007 (0.726- 1.397) | 0.965 | 6 |
| TIMP2 | rs8179090 | C/G | 0.142 | 0.140 | 1.000 | 1.013 (0.666- 1.540) | 0.953 |  |
| SERPINE2 | rs6734100 | G/C | 0.174 | 0.127 | 0.129 | 1.449 (0.953- 2.203) | 0.082 | 7 |
| SERPINE2 | rs729631 | G/C | 0.339 | 0.288 | 0.155 | 1.270 (0.925- 1.744) | 0.139 |  |
| SERPINE2 | rs975278 | A/G | 0.328 | 0.281 | 0.218 | 1.252 (0.910- 1.723) | 0.167 |  |
| SERPINE2 | rs7583463 | A/C | 0.379 | 0.343 | 0.069 | 1.173 (0.865- 1.591) | 0.305 |  |
| SERPINE2 | rs16865421 | G/A | 0.218 | 0.257 | 0.521 | 0.808 (0.574- 1.136) | 0.220 |  |
| SERPINA3 | rs4934 | A/G | 0.436 | 0.414 | 0.609 | 1.094 (0.815- 1.471) | 0.549 | 8 |
| SERPINA3 | rs1800463 | C/T | 0.000 | 0.003 | 1.000 | NA | 0.203 |  |
| SERPINA3 | rs17473 | G/C | 0.002 | 0.000 | 1.000 | NA | 0.431 |  |
| HHIP | rs1828591 | G/A | 0.460 | 0.414 | 0.609 | 1.203 (0.896- 1.615) | 0.220 | 9 |
| HHIP | rs13118928 | G/A | 0.464 | 0.411 | 0.612 | 1.241 (0.924- 1.667) | 0.152 |  |
| CHRNA3/5 | rs8034191 | C/T | 0.193 | 0.216 | 0.811 | 0.868 (0.605- 1.245) | 0.442 | 9 |
| CHRNA3/5 | rs1051730 | T/C | 0.174 | 0.192 | 0.789 | 0.886 (0.608- 1.291) | 0.529 |  |
| IREB2 | rs2568494 | A/G | 0.223 | 0.253 | 0.125 | 0.843 (0.599- 1.186) | 0.326 | 10 |
| IREB2 | rs2656069 | G/A | 0.343 | 0.397 | 0.604 | 0.793 (0.586- 1.072) | 0.131 |  |
| IREB2 | rs1964678 | T/C | 0.477 | 0.490 | 0.868 | 0.949 (0.709- 1.271) | 0.726 |  |
| IREB2 | rs12593229 | T/G | 0.481 | 0.490 | 0.868 | 0.965 (0.721- 1.293) | 0.813 |  |
| IREB2 | rs10851906 | G/A | 0.339 | 0.394 | 1.000 | 0.789 (0.583- 1.068) | 0.125 |  |
| IREB2 | rs965604 | T/C | 0.496 | 0.493 | 1.000 | 1.011 (0.755- 1.353) | 0.944 |  |
| TGF -β | rs1800469 | T/C | 0.350 | 0.404 | 0.392 | 0.793 (0.587- 1.071) | 0.129 | 11 |
| ***IL13*** | **rs1800925** | ***T/C*** | ***0.244*** | ***0.182*** | ***1.000*** | ***1.453 (1.009- 2.091)*** | ***0.044**** | 12 |
| TNF α | rs1800629 | A/G | 0.049 | 0.055 | 0.056 | 0.884 (0.459- 1.702) | 0.711 | 13 |
| IL6 | rs1800795 | C/G | 0.121 | 0.168 | 0.557 | 0.681 (0.451- 1.030) | 0.068 | 14 |
| FAM13A | rs7671167 | T/C | 0.492 | 0.432 | 0.094 | 1.274 (0.950- 1.708) | 0.106 | 15 |
| SFTPB | rs1130866 | T/C | 0.420 | 0.456 | 0.868 | 0.864 (0.644- 1.159) | 0.329 | 16 |
| SFTPD | rs2243639 | T/C | 0.267 | 0.216 | 1.000 | 1.324 (0.937- 1.870) | 0.111 | 16 |
| SFTPD | rs721917 | T/C | 0.290 | 0.267 | 1.000 | 1.122 (0.809- 1.555) | 0.490 |  |
| AQP5 | rs3736309 | G/A | 0.263 | 0.250 | 0.507 | 1.069 (0.765- 1.494) | 0.696 | 17 |
| AQP5 | rs296763 | G/C | 0.051 | 0.058 | 1.000 | 0.867 (0.457- 1.642) | 0.660 |  |
| GC | rs4588 | A/C | 0.263 | 0.281 | 0.839 | 0.913 (0.658- 1.266) | 0.584 | 18 |
| GC | rs7041 | T/G | 0.409 | 0.442 | 0.617 | 0.874 (0.651- 1.174) | 0.371 |  |

*Significant p-values are highlighted in bold italics. The significance was lost after correcting for multiple hypothesis testing by Benjamini–Hochberg False Discovery Rate method.

# Smith CA, Harrison DJ. (1997) Association between polymorphism in gene for microsomal epoxide hydrolase and susceptibility to emphysema. Lancet 350:630–633.

# [Dahl M](http://www.ncbi.nlm.nih.gov/pubmed?term=Dahl%20M%5BAuthor%5D&cauthor=true&cauthor_uid=18703790), [Bowler RP](http://www.ncbi.nlm.nih.gov/pubmed?term=Bowler%20RP%5BAuthor%5D&cauthor=true&cauthor_uid=18703790), [Juul K](http://www.ncbi.nlm.nih.gov/pubmed?term=Juul%20K%5BAuthor%5D&cauthor=true&cauthor_uid=18703790), [Crapo JD](http://www.ncbi.nlm.nih.gov/pubmed?term=Crapo%20JD%5BAuthor%5D&cauthor=true&cauthor_uid=18703790), [Levy S](http://www.ncbi.nlm.nih.gov/pubmed?term=Levy%20S%5BAuthor%5D&cauthor=true&cauthor_uid=18703790), et al. (2008) Superoxide dismutase 3 polymorphism associated with reduced lung function in two large populations. [Am J Respir Crit Care Med.](http://www.ncbi.nlm.nih.gov/pubmed/?term=dahl+M+COPD+SOD3)  178:906-12.

# [Mak JC](http://www.ncbi.nlm.nih.gov/pubmed?term=Mak%20JC%5BAuthor%5D&cauthor=true&cauthor_uid=17567676), [Ho SP](http://www.ncbi.nlm.nih.gov/pubmed?term=Ho%20SP%5BAuthor%5D&cauthor=true&cauthor_uid=17567676), [Yu WC](http://www.ncbi.nlm.nih.gov/pubmed?term=Yu%20WC%5BAuthor%5D&cauthor=true&cauthor_uid=17567676), [Choo KL](http://www.ncbi.nlm.nih.gov/pubmed?term=Choo%20KL%5BAuthor%5D&cauthor=true&cauthor_uid=17567676), [Chu CM](http://www.ncbi.nlm.nih.gov/pubmed?term=Chu%20CM%5BAuthor%5D&cauthor=true&cauthor_uid=17567676), et al. (2007) Polymorphisms and functional activity in superoxide dismutase and catalase genes in smokers with COPD. [Eur Respir J.](http://www.ncbi.nlm.nih.gov/pubmed/17567676) 30:684-90.

# He J-Q, Ruan J, Connett JE, Anthonisen NR, Pare´ PD, et al. (2002) Antioxidant gene polymorphisms and susceptibility to a rapid decline in lung function in smokers. Am J Respir Crit Care Med 166:323–328.

1. [Joos L](http://www.ncbi.nlm.nih.gov/pubmed?term=Joos%20L%5BAuthor%5D&cauthor=true&cauthor_uid=11875051), [He JQ](http://www.ncbi.nlm.nih.gov/pubmed?term=He%20JQ%5BAuthor%5D&cauthor=true&cauthor_uid=11875051), [Shepherdson MB](http://www.ncbi.nlm.nih.gov/pubmed?term=Shepherdson%20MB%5BAuthor%5D&cauthor=true&cauthor_uid=11875051), [Connett JE](http://www.ncbi.nlm.nih.gov/pubmed?term=Connett%20JE%5BAuthor%5D&cauthor=true&cauthor_uid=11875051), [Anthonisen NR](http://www.ncbi.nlm.nih.gov/pubmed?term=Anthonisen%20NR%5BAuthor%5D&cauthor=true&cauthor_uid=11875051), et al. (2002) The role of matrix metalloproteinase polymorphisms in the rate of decline in lung function. [Hum Mol Genet.](http://www.ncbi.nlm.nih.gov/pubmed/11875051?dopt=Abstract) 11: 569-76.

# [Hirano K](http://www.ncbi.nlm.nih.gov/pubmed?term=Hirano%20K%5BAuthor%5D&cauthor=true&cauthor_uid=11757622), [Sakamoto T](http://www.ncbi.nlm.nih.gov/pubmed?term=Sakamoto%20T%5BAuthor%5D&cauthor=true&cauthor_uid=11757622), [Uchida Y](http://www.ncbi.nlm.nih.gov/pubmed?term=Uchida%20Y%5BAuthor%5D&cauthor=true&cauthor_uid=11757622), [Morishima Y](http://www.ncbi.nlm.nih.gov/pubmed?term=Morishima%20Y%5BAuthor%5D&cauthor=true&cauthor_uid=11757622), [Masuyama K](http://www.ncbi.nlm.nih.gov/pubmed?term=Masuyama%20K%5BAuthor%5D&cauthor=true&cauthor_uid=11757622), et al. (2001) Tissue inhibitor of metalloproteinases-2 gene polymorphisms in chronic obstructive pulmonary disease. [Eur Respir J.](http://www.ncbi.nlm.nih.gov/pubmed/11757622) 18: 748-52.

# [Demeo DL](http://www.ncbi.nlm.nih.gov/pubmed?term=Demeo%20DL%5BAuthor%5D&cauthor=true&cauthor_uid=16358219), [Mariani TJ](http://www.ncbi.nlm.nih.gov/pubmed?term=Mariani%20TJ%5BAuthor%5D&cauthor=true&cauthor_uid=16358219), [Lange C](http://www.ncbi.nlm.nih.gov/pubmed?term=Lange%20C%5BAuthor%5D&cauthor=true&cauthor_uid=16358219), [Srisuma S](http://www.ncbi.nlm.nih.gov/pubmed?term=Srisuma%20S%5BAuthor%5D&cauthor=true&cauthor_uid=16358219), [Litonjua AA](http://www.ncbi.nlm.nih.gov/pubmed?term=Litonjua%20AA%5BAuthor%5D&cauthor=true&cauthor_uid=16358219), et al. (2006) The SERPINE2 gene is associated with chronic obstructive pulmonary disease. [Am J Hum Genet.](http://www.ncbi.nlm.nih.gov/pubmed/16358219) 78: 253-64.

# [Ishii T](http://www.ncbi.nlm.nih.gov/pubmed?term=Ishii%20T%5BAuthor%5D&cauthor=true&cauthor_uid=10849024), [Matsuse T](http://www.ncbi.nlm.nih.gov/pubmed?term=Matsuse%20T%5BAuthor%5D&cauthor=true&cauthor_uid=10849024), [Teramoto S](http://www.ncbi.nlm.nih.gov/pubmed?term=Teramoto%20S%5BAuthor%5D&cauthor=true&cauthor_uid=10849024), [Matsui H](http://www.ncbi.nlm.nih.gov/pubmed?term=Matsui%20H%5BAuthor%5D&cauthor=true&cauthor_uid=10849024), [Hosoi T](http://www.ncbi.nlm.nih.gov/pubmed?term=Hosoi%20T%5BAuthor%5D&cauthor=true&cauthor_uid=10849024), et al. (2000) Association between alpha-1-antichymotrypsin polymorphism and susceptibility to chronic obstructive pulmonary disease. [Eur J Clin Invest.](http://www.ncbi.nlm.nih.gov/pubmed/10849024?dopt=Abstract) 30: 543-8.

# [Pillai SG](http://www.ncbi.nlm.nih.gov/pubmed?term=Pillai%20SG%5BAuthor%5D&cauthor=true&cauthor_uid=19300482), [Ge D](http://www.ncbi.nlm.nih.gov/pubmed?term=Ge%20D%5BAuthor%5D&cauthor=true&cauthor_uid=19300482), [Zhu G](http://www.ncbi.nlm.nih.gov/pubmed?term=Zhu%20G%5BAuthor%5D&cauthor=true&cauthor_uid=19300482), [Kong X](http://www.ncbi.nlm.nih.gov/pubmed?term=Kong%20X%5BAuthor%5D&cauthor=true&cauthor_uid=19300482), [Shianna KV](http://www.ncbi.nlm.nih.gov/pubmed?term=Shianna%20KV%5BAuthor%5D&cauthor=true&cauthor_uid=19300482), et al. (2009) A genome-wide association study in chronic obstructive pulmonary disease (COPD): identification of two major susceptibility loci. [PLoS Genet.](http://www.ncbi.nlm.nih.gov/pubmed/19300482) 5: e1000421.

# [DeMeo DL](http://www.ncbi.nlm.nih.gov/pubmed?term=DeMeo%20DL%5BAuthor%5D&cauthor=true&cauthor_uid=19800047), [Mariani T](http://www.ncbi.nlm.nih.gov/pubmed?term=Mariani%20T%5BAuthor%5D&cauthor=true&cauthor_uid=19800047), [Bhattacharya S](http://www.ncbi.nlm.nih.gov/pubmed?term=Bhattacharya%20S%5BAuthor%5D&cauthor=true&cauthor_uid=19800047), [Srisuma S](http://www.ncbi.nlm.nih.gov/pubmed?term=Srisuma%20S%5BAuthor%5D&cauthor=true&cauthor_uid=19800047), [Lange C](http://www.ncbi.nlm.nih.gov/pubmed?term=Lange%20C%5BAuthor%5D&cauthor=true&cauthor_uid=19800047), et al. (2009) Integration of genomic and genetic approaches implicates IREB2 as a COPD susceptibility gene. [Am J Hum Genet.](http://www.ncbi.nlm.nih.gov/pubmed/19800047) 85: 493-502.

# Wu L, Chau J, Young RP, Pokorny V, Mills GD, et al. (2004) Transforming growth factor-beta1 genotype and susceptibilityto chronic obstructive pulmonary disease. Thorax 59: 126–129.

# [van der Pouw Kraan TC](http://www.ncbi.nlm.nih.gov/pubmed?term=van%20der%20Pouw%20Kraan%20TC%5BAuthor%5D&cauthor=true&cauthor_uid=12424628), [Küçükaycan M](http://www.ncbi.nlm.nih.gov/pubmed?term=K%C3%BC%C3%A7%C3%BCkaycan%20M%5BAuthor%5D&cauthor=true&cauthor_uid=12424628), [Bakker AM](http://www.ncbi.nlm.nih.gov/pubmed?term=Bakker%20AM%5BAuthor%5D&cauthor=true&cauthor_uid=12424628), [Baggen JM](http://www.ncbi.nlm.nih.gov/pubmed?term=Baggen%20JM%5BAuthor%5D&cauthor=true&cauthor_uid=12424628), [van der Zee JS](http://www.ncbi.nlm.nih.gov/pubmed?term=van%20der%20Zee%20JS%5BAuthor%5D&cauthor=true&cauthor_uid=12424628), et al. (2002) Chronic obstructive pulmonary disease is associated with the -1055 IL-13 promoter polymorphism. [Genes Immun.](http://www.ncbi.nlm.nih.gov/pubmed/12424628?dopt=Abstract) 3: 436-9.

# [Sakao S](http://www.ncbi.nlm.nih.gov/pubmed?term=Sakao%20S%5BAuthor%5D&cauthor=true&cauthor_uid=11179116), [Tatsumi K](http://www.ncbi.nlm.nih.gov/pubmed?term=Tatsumi%20K%5BAuthor%5D&cauthor=true&cauthor_uid=11179116), [Igari H](http://www.ncbi.nlm.nih.gov/pubmed?term=Igari%20H%5BAuthor%5D&cauthor=true&cauthor_uid=11179116), [Shino Y](http://www.ncbi.nlm.nih.gov/pubmed?term=Shino%20Y%5BAuthor%5D&cauthor=true&cauthor_uid=11179116), [Shirasawa H](http://www.ncbi.nlm.nih.gov/pubmed?term=Shirasawa%20H%5BAuthor%5D&cauthor=true&cauthor_uid=11179116), et al. (2001) Association of tumor necrosis factor alpha gene promoter polymorphism with the presence of chronic obstructive pulmonary disease. [Am J Respir Crit Care Med.](http://www.ncbi.nlm.nih.gov/pubmed/11179116?dopt=Abstract) 163: 420-2.

# [He JQ](http://www.ncbi.nlm.nih.gov/pubmed?term=He%20JQ%5BAuthor%5D&cauthor=true&cauthor_uid=19359268), [Foreman MG](http://www.ncbi.nlm.nih.gov/pubmed?term=Foreman%20MG%5BAuthor%5D&cauthor=true&cauthor_uid=19359268), [Shumansky K](http://www.ncbi.nlm.nih.gov/pubmed?term=Shumansky%20K%5BAuthor%5D&cauthor=true&cauthor_uid=19359268), [Zhang X](http://www.ncbi.nlm.nih.gov/pubmed?term=Zhang%20X%5BAuthor%5D&cauthor=true&cauthor_uid=19359268), [Akhabir L](http://www.ncbi.nlm.nih.gov/pubmed?term=Akhabir%20L%5BAuthor%5D&cauthor=true&cauthor_uid=19359268), et al. (2009) Associations of IL6 polymorphisms with lung function decline and COPD. [Thorax.](http://www.ncbi.nlm.nih.gov/pubmed/19359268) 64: 698-704.

# Young RP, Hopkins RJ, Hay BA, Whittington CF, Epton MJ, et al. (2010) FAM13A locus in COPD is independently associated with lung cancer - evidence of a molecular genetic link between COPD and lung cancer. Appl Clin Genet. 4: 1-10.

# [Hersh CP](http://www.ncbi.nlm.nih.gov/pubmed?term=Hersh%20CP%5BAuthor%5D&cauthor=true&cauthor_uid=15817713), [Demeo DL](http://www.ncbi.nlm.nih.gov/pubmed?term=Demeo%20DL%5BAuthor%5D&cauthor=true&cauthor_uid=15817713), [Lange C](http://www.ncbi.nlm.nih.gov/pubmed?term=Lange%20C%5BAuthor%5D&cauthor=true&cauthor_uid=15817713), [Litonjua AA](http://www.ncbi.nlm.nih.gov/pubmed?term=Litonjua%20AA%5BAuthor%5D&cauthor=true&cauthor_uid=15817713), [Reilly JJ](http://www.ncbi.nlm.nih.gov/pubmed?term=Reilly%20JJ%5BAuthor%5D&cauthor=true&cauthor_uid=15817713), et al. (2005) Attempted replication of reported chronic obstructive pulmonary disease candidate gene associations. [Am J Respir Cell Mol Biol.](http://www.ncbi.nlm.nih.gov/pubmed/15817713?dopt=Abstract) 33: 71-8.

# [Hansel NN](http://www.ncbi.nlm.nih.gov/pubmed?term=Hansel%20NN%5BAuthor%5D&cauthor=true&cauthor_uid=21151978), [Sidhaye V](http://www.ncbi.nlm.nih.gov/pubmed?term=Sidhaye%20V%5BAuthor%5D&cauthor=true&cauthor_uid=21151978), [Rafaels NM](http://www.ncbi.nlm.nih.gov/pubmed?term=Rafaels%20NM%5BAuthor%5D&cauthor=true&cauthor_uid=21151978), [Gao L](http://www.ncbi.nlm.nih.gov/pubmed?term=Gao%20L%5BAuthor%5D&cauthor=true&cauthor_uid=21151978), [Gao P](http://www.ncbi.nlm.nih.gov/pubmed?term=Gao%20P%5BAuthor%5D&cauthor=true&cauthor_uid=21151978), et al. (2010) Aquaporin 5 polymorphisms and rate of lung function decline in chronic obstructive pulmonary disease. [PLoS One.](http://www.ncbi.nlm.nih.gov/pubmed/21151978) 5: e14226.

1. Schellenberg D, Pare PD, Weir TD, Spinelli JJ, Walker BA, et al. (1998) Vitamin D binding protein variants and the risk of COPD. Am J Respir Crit Care Med 157: 957-961.
