# Supplementary material for: Genetic Determinants of Chronic Obstructive Pulmonary Disease in South Indian Male Smokers
Source: PLoS One. 2014 Feb 24;9(2):e89957. doi: 10.1371/journal.pone.0089957 (PMC3933698; doi:10.1371/journal.pone.0089957)
Supplement: Table S2 — (DOCX) [file pone.0089957.s002.docx]

Supplementary table 2. Results of genotype association analysis under different genetic models.

| **GENE** | **SNP** | **ADDITIVE** | | **DOMINANT** | | **RECESSIVE** | |
| --- | --- | --- | --- | --- | --- | --- | --- |
|  |  | **OR (95%CI)** | **P*** | **OR (95%CI)** | **P*** | **OR (95%CI)** | **P*** |
| EPHX1 | rs1051740 | 0.929(0.686-1.260) | 0.637 | 0.733(0.471-1.141) | 0.169 | 1.327(0.731-2.409) | 0.353 |
| EPHX1 | rs2234922 | 0.900(0.636-1.275) | 0.555 | 0.921(0.596-1.423) | 0.710 | 0.710(0.291-1.730) | 0.451 |
| SOD3 | rs8192287 | 1.047(0.667-1.646) | 0.841 | 1.006(0.621-1.631) | 0.980 | 2.433(0.248-23.910) | 0.446 |
| SOD3 | rs8192288 | 1.026(0.653-1.614) | 0.911 | 0.983(0.606-1.595) | 0.944 | 2.433(0.248-23.910) | 0.446 |
| SOD3 | rs1799895 | 1.279(0.658-2.487) | 0.468 | 1.279(0.658-2.487) | 0.468 | NA |  |
| CAT | rs1001179 | 1.021(0.712-1.466) | 0.909 | 1.017(0.668-1.549) | 0.936 | 1.070(0.382-2.997) | 0.898 |
| ***GSTP1*** | ***rs1695*** | 1.168(0.836-1.631) | 0.364 | 0.994(0.653-1.512) | 0.977 | ***2.756(1.081-7.026)*** | ***0.034*** |
| GSTP1 | rs1138272 | 0.682(0.363-1.278) | 0.232 | 0.612(0.302-1.241) | 0.173 | 1.081(0.096-12.150) | 0.950 |
| MMP12 | rs652438 | 0.845(0.480-1.489) | 0.561 | 0.893(0.479-1.664) | 0.722 | 0.291(0.026-3.298) | 0.319 |
| ***MMP12*** | ***rs2276109*** | ***0.478(0.261-0.875)*** | ***0.017*** | ***0.443(0.234-0.838)*** | ***0.012*** | 0.648(0.040-10.560) | 0.760 |
| TIMP2 | rs2277698 | 0.992(0.715-1.377) | 0.963 | 1.031(0.677-1.571) | 0.888 | 0.868(0.405-1.857) | 0.714 |
| TIMP2 | rs8179090 | 1.037(0.672-1.598) | 0.871 | 1.071(0.666-1.723) | 0.777 | 0.735(0.142-3.806) | 0.714 |
| SERPINE2 | rs6734100 | 1.462(0.944-2.263) | 0.089 | 1.323(0.821-2.131) | 0.250 | NA | 0.998 |
| ***SERPINE2*** | ***rs729631*** | 1.245(0.901-1.721) | 0.184 | 1.079(0.709-1.644) | 0.722 | ***2.558(1.131-5.788)*** | ***0.024*** |
| ***SERPINE2*** | ***rs975278*** | 1.219(0.884-1.680) | 0.228 | 1.044(0.686-1.590) | 0.840 | ***2.558(1.131-5.788)*** | ***0.024*** |
| ***SERPINE2*** | ***rs7583463*** | 1.122(0.828-1.520) | 0.458 | 0.845(0.551-1.298) | 0.442 | ***2.407(1.212-4.781)*** | ***0.012*** |
| SERPINE2 | rs16865421 | 0.805(0.575-1.125) | 0.204 | 0.707(0.457-1.092) | 0.118 | 0.945(0.423-2.111) | 0.890 |
| SERPINA3 | rs4934 | 1.062(0.786-1.435) | 0.696 | 0.950(0.607-1.488) | 0.824 | 1.321(0.756-2.311) | 0.329 |
| HHIP | rs1828591 | 1.142(0.854-1.526) | 0.371 | 0.945(0.604-1.478) | 0.805 | 1.674(0.975-2.877) | 0.062 |
| HHIP | rs13118928 | 1.176(0.879-1.574) | 0.275 | 1.013(0.648-1.584) | 0.955 | 1.674(0.975-2.877) | 0.062 |
| CHRNA3/5 | rs8034191 | 0.870(0.596-1.271) | 0.472 | 0.903(0.586-1.391) | 0.642 | 0.556(0.172-1.800) | 0.328 |
| CHRNA3/5 | rs1051730 | 0.893(0.607-1.311) | 0.562 | 0.934(0.599-1.456) | 0.763 | 0.556(0.172-1.800) | 0.328 |
| ***IREB2*** | ***rs2568494*** | 0.878(0.618-1.248) | 0.469 | 1.040(0.679-1.594) | 0.857 | ***0.336(0.129-0.876)*** | ***0.026*** |
| IREB2 | rs2656069 | 0.751(0.543-1.039) | 0.084 | 0.771(0.499-1.191) | 0.242 | 0.557(0.290-1.070) | 0.079 |
| IREB2 | rs1964678 | 0.937(0.693-1.266) | 0.671 | 0.913(0.566-1.473) | 0.709 | 0.922(0.558-1.524) | 0.752 |
| IREB2 | rs12593229 | 0.948(0.701-1.282) | 0.729 | 0.927(0.574-1.496) | 0.755 | 0.938(0.568-1.549) | 0.803 |
| ***IREB2*** | ***rs10851906*** | 0.765(0.556-1.052) | 0.099 | 0.825(0.536-1.269) | 0.381 | ***0.512(0.270-0.972)*** | ***0.041*** |
| IREB2 | rs965604 | 1.024(0.760-1.380) | 0.876 | 1.012(0.623-1.644) | 0.961 | 1.053(0.646-1.716) | 0.837 |
| TGF -β | rs1800469 | 0.780(0.572-1.065) | 0.118 | 0.683(0.441-1.057) | 0.087 | 0.815(0.441-1.503) | 0.511 |
| ***IL13*** | ***rs1800925*** | ***1.443(1.005-2.071)*** | ***0.047*** | 1.461(0.941-2.269) | 0.092 | 2.306(0.833-6.384) | 0.108 |
| TNF α | rs1800629 | 0.912(0.477-1.741) | 0.779 | 1.048(0.515-2.133) | 0.896 | NA | 0.999 |
| IL6 | rs1800795 | 0.678(0.446-1.029) | 0.068 | 0.660(0.410-1.064) | 0.088 | 0.472(0.122-1.830) | 0.277 |
| ***FAM13A*** | ***rs7671167*** | 1.302(0.956-1.774) | 0.094 | 1.151(0.719-1.844) | 0.558 | ***1.853(1.066-3.223)*** | ***0.029*** |
| SFTPB | rs1130866 | 0.859(0.640-1.152) | 0.309 | 0.803(0.512-1.261) | 0.341 | 0.829(0.492-1.399) | 0.483 |
| SFTPD | rs2243639 | 1.228(0.858-1.757) | 0.261 | 1.305(0.850-2.005) | 0.224 | 1.162(0.455-2.965) | 0.754 |
| SFTPD | rs721917 | 1.061(0.757-1.487) | 0.730 | 1.082(0.709-1.651) | 0.716 | 1.057(0.471-2.370) | 0.894 |
| AQP5 | rs3736309 | 1.096(0.774-1.551) | 0.607 | 1.015(0.664-1.552) | 0.945 | 1.739(0.676-4.472) | 0.251 |
| AQP5 | rs296763 | 0.822(0.419-1.612) | 0.569 | 0.822(0.419-1.612) | 0.569 | NA |  |
| GC | rs4588 | 0.964(0.690-1.346) | 0.828 | 0.992(0.650-1.514) | 0.969 | 0.832(0.374-1.850) | 0.651 |
| GC | rs7041 | 0.945(0.699-1.277) | 0.711 | 0.987(0.630-1.546) | 0.955 | 0.846(0.491-1.456) | 0.546 |

* Significant p-values are highlighted in bold italics. P- values have been adjusted for age and pack years. Significance was lost after correcting for multiple hypothesis testing by Benjamini–Hochberg False Discovery Rate method.
