# Supplementary material for: Genetic Determinants of Chronic Obstructive Pulmonary Disease in South Indian Male Smokers
Source: PLoS One. 2014 Feb 24;9(2):e89957. doi: 10.1371/journal.pone.0089957 (PMC3933698; doi:10.1371/journal.pone.0089957)
Supplement: Table S3 — (DOCX) [file pone.0089957.s003.docx]

**Supplementary table 3. Results of haplotype association analysis.**

| **SNP1** | **SNP2** | **SNP3** | **SNP4** | **HAPLOTYPE** | **f A** | **f U** | **OR** | **P*COPD** |
| --- | --- | --- | --- | --- | --- | --- | --- | --- |
| rs652438 | rs2276109 |  |  | AG | 0.044 | 0.089 | 0.478 | 0.017 |
| rs652438 | rs2276109 |  |  | AA | 0.894 | 0.836 | 1.6 | 0.031 |
| rs2568494 | rs2656069 |  |  | GA | 0.434 | 0.349 | 1.41 | 0.028 |
| rs2656069 | rs1964678 |  |  | AT | 0.143 | 0.092 | 1.64 | 0.044 |
| rs975278 | rs7583463 | rs16865421 |  | GCG | 0.059 | 0.098 | 0.549 | 0.048 |
| rs1138272 | rs652438 | rs2276109 |  | CAG | 0.044 | 0.086 | 0.489 | 0.023 |
| rs1138272 | rs652438 | rs2276109 |  | CAA | 0.857 | 0.787 | 1.61 | 0.014 |
| rs2568494 | rs2656069 | rs1964678 |  | GAT | 0.140 | 0.085 | 1.73 | 0.030 |
| rs2656069 | rs1964678 | rs12593229 |  | ATT | 0.143 | 0.092 | 1.64 | 0.044 |
| rs4588 | rs7041 | rs7671167 | rs1828591 | CTCA | 0.028 | 0.056 | 0.294 | 0.022 |
| rs1695 | rs1138272 | rs652438 | rs2276109 | ACAG | 0.038 | 0.069 | 0.488 | 0.047 |
| rs2568494 | rs2656069 | rs1964678 | rs12593229 | GATT | 0.140 | 0.085 | 1.73 | 0.030 |
| rs2656069 | rs1964678 | rs12593229 | rs10851906 | ATTA | 0.144 | 0.091 | 1.68 | 0.036 |
|  |  |  |  |  |  |  |  |  |
| **SNP1** | **SNP2** | **SNP3** | **SNP4** | **HAPLOTYPE** | **BETA** | **P*FEV_1_** |  |  |
| rs7041 | rs7671167 |  |  | GT | -3.85 | 0.051 |  |  |
| rs7041 | rs7671167 |  |  | TC | 4.18 | 0.025 |  |  |
| rs7671167 | rs1828591 |  |  | CA | 4.17 | 0.023 |  |  |
| rs1695 | rs1138272 |  |  | GC | -4.59 | 0.010 |  |  |
| rs1695 | rs1138272 |  |  | AC | 3.43 | 0.043 |  |  |
| rs2568494 | rs2656069 |  |  | GA | -3.37 | 0.028 |  |  |
| rs7041 | rs7671167 | rs1828591 |  | TCA | 6.22 | 0.018 |  |  |
| rs7671167 | rs1828591 | rs13118928 |  | CAA | 4.18 | 0.023 |  |  |
| rs1001179 | rs1695 | rs1138272 |  | GGC | -4.89 | 0.023 |  |  |
| rs1695 | rs1138272 | rs652438 |  | GCA | -4.41 | 0.015 |  |  |
| rs1799895 | rs4588 | rs7041 | rs7671167 | CCGT | -4.25 | 0.038 |  |  |
| rs4588 | rs7041 | rs7671167 | rs1828591 | CTCA | 12.1 | 0.024 |  |  |
| rs7041 | rs7671167 | rs1828591 | rs13118928 | TCAA | 6.01 | 0.021 |  |  |
| rs1001179 | rs1695 | rs1138272 | rs652438 | GGCA | -4.61 | 0.038 |  |  |
| rs1695 | rs1138272 | rs652438 | rs2276109 | GCAA | -5.23 | 0.005 |  |  |
|  |  |  |  |  |  |  |  |  |
| **SNP1** | **SNP2** | **SNP3** | **SNP4** | **HAPLOTYPE** | **BETA** | **P*FEV_1_/FVC** |  |  |
| rs1051740 | rs2234922 |  |  | CG | 6.44 | 0.037 |  |  |
| rs7583463 | rs16865421 |  |  | CG | 5.8 | 0.018 |  |  |
| rs652438 | rs2276109 |  |  | AG | 6.02 | 0.016 |  |  |
| rs2568494 | rs2656069 |  |  | GA | -3.34 | 0.006 |  |  |
| rs2656069 | rs1964678 |  |  | AT | -4.03 | 0.025 |  |  |
| rs12593229 | rs10851906 |  |  | TA | -3.63 | 0.040 |  |  |
| rs975278 | rs7583463 | rs16865421 |  | GCG | 5.87 | 0.017 |  |  |
| rs7041 | rs7671167 | rs1828591 |  | TCA | 4.3 | 0.039 |  |  |
| rs1138272 | rs652438 | rs2276109 |  | CAG | 5.88 | 0.021 |  |  |
| rs1138272 | rs652438 | rs2276109 |  | CAA | -3.26 | 0.037 |  |  |
| rs2568494 | rs2656069 | rs1964678 |  | GAT | -4.35 | 0.017 |  |  |
| rs2656069 | rs1964678 | rs12593229 |  | ATT | -4.03 | 0.025 |  |  |
| rs1964678 | rs12593229 | rs10851906 |  | TTA | -3.63 | 0.040 |  |  |
| rs12593229 | rs10851906 | rs965604 |  | TAC | -3.63 | 0.040 |  |  |
| rs729631 | rs975278 | rs7583463 | rs16865421 | CGCG | 6.01 | 0.019 |  |  |
| rs4588 | rs7041 | rs7671167 | rs1828591 | CTCA | 8.75 | 0.041 |  |  |
| rs7041 | rs7671167 | rs1828591 | rs13118928 | TCAA | 4.28 | 0.038 |  |  |
| rs1695 | rs1138272 | rs652438 | rs2276109 | GCAG | 16.2 | 0.035 |  |  |
| rs2568494 | rs2656069 | rs1964678 | rs12593229 | GATT | -4.35 | 0.017 |  |  |
| rs2656069 | rs1964678 | rs12593229 | rs10851906 | ATTA | -4.19 | 0.020 |  |  |
| rs1964678 | rs12593229 | rs10851906 | rs965604 | TTAC | -3.63 | 0.040 |  |  |
| rs12593229 | rs10851906 | rs965604 | rs8034191 | TACT | -3.63 | 0.040 |  |  |

P*: p-value adjusted for age and pack years. None of the haplotypes retained significance after adjusting for multiple testing.

f A and f U: Frequency of haplotype in affected and unaffected respectively.
